# BURDENS OF HONOR: EXAMINING HONOR IDEOLOGY, SUICIDE RISK-FACTORS, AND AGEISM IN OLDER ADULTS

**DOI:** 10.1093/geroni/igz038.1138

**Published:** 2019-11-08

**Authors:** Jarrod Bock, Ryan P Brown

**Affiliations:** 1 Oklahoma State University, Stillwater, Oklahoma, United States; 2 Rice University, Houston, Texas, United States

## Abstract

Prior research has demonstrated that rates of suicide increase as men enter older adulthood and that these rates are even higher in honor-oriented regions of the U.S. (particularly among White men). Research into the honor-suicide link has suggested explanatory factors that coincide with the interpersonal theory of suicide, such as untreated depression, heightened risk-taking, and the use of firearms in suicide; however, factors related to aging (e.g., ageism, anxiety about aging) have yet to be examined. The present study examined ambivalent ageism, permissive attitudes toward suicide, and interpersonal risk-factors for suicide as explanations for the honor-suicide link among a sample of 201 older American men (Mage = 56.45, SD = 8.35, range = 44-77 years of age). After controlling for participant age and religiosity, participants with greater endorsement of honor ideology but lower levels of honor fulfillment expressed heightened levels of thwarted belongingness (β = 0.35, p = .001)—an established interpersonal risk-factor for suicide. Additionally, lower levels of honor fulfillment predicted greater anxiety about aging (β = -0.41, p < .001), greater perceived burdensomeness (β = -0.39, p < .001), and more positive implicit attitudes toward youth (β = 0.27, p = .019). Conversely, greater levels of honor fulfillment predicted more positive attitudes toward aging (β = 0.20, p = .025). Our results extend previous research on the honor-suicide relationship by demonstrating the utility of marrying the interpersonal theory of suicide with research on cultures of honor.

